# Acylglycerol kinase promotes ovarian cancer progression and regulates mitochondria function by interacting with ribosomal protein L39

**DOI:** 10.1186/s13046-022-02448-5

**Published:** 2022-08-08

**Authors:** Fei Sun, Yunjian Wei, Zheng Liu, Qiuling Jie, Xiaohui Yang, Ping Long, Jun Wang, Ying Xiong, Qi Li, Song Quan, Yanlin Ma

**Affiliations:** 1 Department of Obstetrics and Gynecology, Reproductive Medicine, Nanfang Hospital, Southern Medical University, Guangzhou, 510515 Guangdong China; 2 Hainan Provincial Key Laboratory for Human Reproductive Medicine and Genetic Research, Hainan Provincial Clinical Research Center for Thalassemia, the Key Laboratory of Tropical Translational Medicine of Ministry of Education, Department of Reproductive Medicine, the First Affiliated Hospital of Hainan Medical University, Hainan Medical University, Haikou, 571101 Hainan China; 3 Haikou Key Laboratory for Preservation of Human Genetic Resource, the First Affiliated Hospital of Hainan Medical University, Haikou, 571101 Hainan China; 4 College of Medical Laboratory Science, Guilin Medical University, Guilin, 541004 Guangxi China; 5 Texas Heart Institute, Houston, TX 77030 USA; 6 Department of Gynecologic Oncology, Sun Yat-sen University Cancer Center, Guangzhou, Guangdong 510060 China; 7 State Key Laboratory of Oncology in South China, Guangzhou, Guangdong 510060, China; 8 Collaborative Innovation Center of Cancer Medicine, Guangzhou, Guangdong 510060 China; 9 Hainan Modern Women and Children’s Hospital, Reproductive Medicine, Haikou, 571199 Hainan China

**Keywords:** Epithelial ovarian cancer, Acylglycerol kinase, Ribosomal protein L39, cancer stem cell, Mitochondrial

## Abstract

**Background:**

Epithelial ovarian cancer (EOC) is the leading cause of deaths among patients with gynecologic malignancies. In recent years, cancer stem cells (CSCs) have attracted great attention, which have been regarded as new biomarkers and targets in cancer diagnoses as well as therapies. However, therapeutic failure caused by chemotherapy resistance in late-stage EOC occurs frequently. The 5-year survival rate of patients with EOC remains at about 30%.

**Methods:**

In this study, the expression of acylglycerol kinase (AGK) was analyzed among patients with EOC. The effect of AGK on EOC cell proliferation and tumorigenicity was studied using Western blotting, flow cytometry, EdU assay and in vivo xenotransplantation assays. Furthermore, AGK induced CSC-like properties and was resistant to cisplatin chemotherapy in the EOC cells, which were investigated through sphere formation assays and the in vivo model of chemoresistance. Finally, the relationship between AGK and RPL39 (Ribosomal protein L39) in mitochondria as well as their effect on the mitochondrial function was analyzed through methods including transmission electron microscopy, microarray, biotin identification and immunoprecipitation.

**Results:**

AGK showed a markedly upregulated expression in EOC, which was significantly associated with the poor survival of patients with EOC, the expression of AGK-promoted EOC cell proliferation and tumorigenicity. AGK also induced CSC-like properties in the EOC cells and was resistant to cisplatin chemotherapy. Furthermore, the results indicated that AGK not only maintained mitochondrial cristae morphogenesis, but also increased the production of reactive oxygen species and Δψm of EOC cells in a kinase-independent manner. Finally, our results revealed that AGK played its biological function by directly interacting with RPL39.

**Conclusions:**

We demonstrated that AGK was a novel CSC biomarker for EOC, which the stemness of EOC was promoted and chemotherapy resistance was developed through physical as well as functional interaction with RPL39.

**Supplementary Information:**

The online version contains supplementary material available at 10.1186/s13046-022-02448-5.

## Background

Epithelial ovarian cancer (EOC) accounts for approximately 90% of all ovarian cancers, which is the leading cause of deaths among patients with gynecologic malignant tumors [1, 2]. According to previous studies, the lack of reliable early diagnostic methods resulted in 70% of patients with EOC being diagnosed in its late stage [3]. Despite the rapid development of EOC detection technology in recent years, the 5-year survival rate of patients with EOC remains at about 30% [4]. Accumulated evidences have suggested that cancer stem cells (CSCs) are responsible for tumor progression, chemoresistance and recurrence [5, 6]. CSC markers, including ALDH1, CD44, SOX2, Oct4 and Lin28, have been shown to be reliable predictors for breast cancer, lung cancer and pancreas cancer [7, 8], but those for EOC remain unclear. Therefore, identifying specific CSC markers for EOC becomes a responsibility of present-day research on the cancer [9].

Acylglycerol kinase (AGK) is a newly discovered mitochondrial lipid kinase that catalyzes the phosphorylation of monoacylglycerol and diacylglycerol, so as to produce lysophosphatidic acid and phosphatidic acid respectively [10, 11]. AGK has been shown to be a significant cancer-related gene, which is overexpressed in multiple cancers and has been found to enhance the tumor-initiating ability of prostate cancer cells [12]. The overexpression of AGK activates the JAK2/STAT3 pathway, consequently increasing the self-renewal ability of CSCs and the tumorigenicity of esophageal squamous carcinoma cells [13]. AGK also promotes the growth of renal cell carcinomas and exacerbates cancer progression via activating the PI3K/AKT/GSK3β signaling pathway [14]. In this study, we found that AGK promoted the proliferation and tumorigenicity of EOC both in vivo and in vitro. The overexpression of AGK promoted CSC-like characteristics in the EOC cells and was resistant to cisplatin chemotherapy. Furthermore, our results revealed that AGK affected the mitochondrial structure in a kinase-independent manner and directly interacted with RPL39 (ribosomal protein L39).

## Materials and methods

### Patient information and human tissue preparation

All patients with EOC involved in this study were histologically confirmed, who received treatment at the Sun Yat-sen University Cancer Center from January 2003 to December 2008. Patient medical records were reviewed to obtain demographic and clinical data, including age, the level of serum CA125, diagnosis, the volume of ascites, surgical procedures, tumor stage, pathological reports, post-operation chemotherapy and patients’ follow-up results. These patients had not been treated with radiotherapy or chemotherapy before surgery. Clinical follow-up data was available until December 31, 2016. The clinical information on these patients is summarized in Table 1.

**Table 1:** The correlations between AGK expression and the clinicopathological features of the patients with epithelial ovarian cancer

Characteristics	Number of cases (%)	AGK expression (%)		*P* value
		Low or no expression	High expression	
Age (years)				0.710
<45	49 (35)	18 (36.7)	31 (63.2)	
≥ 45	91 (65)	30 (33.0)	61 (67.0)	
CA125 level				0.482
<500 U/ml	67 (47.9)	25 (37.3)	42 (62.7)	
≥ 500 U/ml	73 (52.1)	23 (31.5)	50 (68.5)	
Tumor size				0.206
<10 cm	58 (41.4)	16 (27.6)	42 (72.4)	
≥ 10 cm	82 (58.6)	32 (39.0)	50 (61.0)	
The volume of ascites				**0.001**
<1000 ml	100 (71.4)	43 (43.0)	57 (57.0)	
≥ 1000 ml	40 (28.6)	5 (12.5)	35 (87.5)	
Peritoneal cytology				**0.004**
Positive	68 (48.6)	15 (22.0)	53 (78.0)	
Negative	72 (51.4)	33 (45.8)	39 (54.2)	
Pathological type				0.238
Serous	80 (57.1)	25 (31.2)	55 (68.8)	
Mucinous	14 (10.0)	8 (57.1)	6 (42.9)	
Poorly differentiated	36 (25.7)	12 (33.3)	24 (66.7)	
Others^a^	10 (7.1)	3 (30.0)	7 (70.0)	
Grade of differentiation				0.376
G1	59 (42.1)	23 (39.0)	36 (61.0)	
G2	46 (32.9)	14 (30.4)	32 (69.6)	
G3	18 (12.9)	6 (33.3)	12 (66.7)	
Unknown	17 (12.1)	5 (29.4)	12 (70.6)	
FIGO stage				**0.002**
I	30 (21.4)	18 (60.0)	12 (30.0)	
II+ III + IV	110 (78.6)	32 (29.1)	80 (70.9)	
Lymph node metastasis				0.688
Positive	21 (15.0)	8 (38.1)	13 (61.9)	
Negative	23 (16.4)	7 (30.4)	16 (69.6)	
Not do RPLND^b^	96 (68.6)	33 (34.4)	63 (65.6)	
Cytoreductive surgery^c^				0.812
Optimal	107 (76.4)	35 (32.7)	72 (67.3)	
Suboptimal	33 (23.6)	13 (39.4)	20 (60.6)	

^a^Endometrioid adenocarcinoma 2 cases, clear cell carcinoma 3 cases, mixed epithelial carcinoma 5 cases

^b^RPLND, retroperitoneal lymph node dissection, including unilateral or bilateral pelvic lymphadenectomy and /or paraortic lymphadenectomy

^c^Cytoreductive surgery: Optimal, the diameter of biggest residual lesions < 2 cm；Suboptimal, the diameter of biggest residual lesions ≥2 cm

***P < 0.05*** was considered statistically significant

Ten matched pairs of fresh tumor tissue specimens (T) and adjacent noncancerous tissue (ANT) samples were obtained from patients with EOC after surgery and were snap-frozen at − 80 °C until use. Percentage tumor purity of tumor tissues and adjacent sections used for RNA and protein extraction was estimated through routine histopathologic analysis to assure that the sections contained major cancer lesions. A total of 140 EOC tissue samples and 20 normal ovarian epithelial tissues collected from patients undergoing benign hysterectomy and oophorectomy were analyzed through immunohistochemistry (IHC).

### Cell lines

HEK293, SKOV3, ES-2 and HO8910 cells were purchased from Shanghai Cell Bank of the Chinese Academy of Science (Shanghai, China). SKOV-3 and ES-2 cells were grown in McCoy’s 5A medium supplemented with 10% fetal bovine serum (FBS). HEK293 and HO8910 cells were grown in Dulbecco’s modified Eagle medium (DMEM) - high glucose medium (Life, U.S.) supplemented with 10% FBS (Life, U.S.). Ovarian cancer cell lines (OVCAR3) and Anglne cells were purchased from China Center for Type Culture Collection (Wuhan, China). OVCAR3 cells and Anglne cells were respectively grown in DMEM-high glucose medium with 10% FBS and Eagle’s minimal essential medium (Eagle’s MEM) with 10% FBS. A2780 cells were purchased from Nanjing KeyGen Biotech (Nanjing, China) and cultured in a DMEM-high glucose medium with 10% FBS. Primary normal ovarian surface epithelial (NOSE) cells were established through the previously reported methods [15]. All cell lines were maintained in 5% CO_2_ at 37 °C.

### Plasmids, virus production and infection

The human AGK gene was amplified and subcloned into the *XhoI* and *BamHI* site of a pLVX-AcGFP1-N1 lentiviral vector (Clontech, U.S.). shRNA-targeting AGK was subcloned into the *AgeI* and *EcoRI* site of a GV248-EGFP-puromycin lentiviral vector (GENE, China)*.* The human RPL39 gene was subcloned into the *EcoRI* and *BamHI* site of a pSin-EF1α-purolentiviral vector (donated by GuangZhou Institutes of Biomedicine and Health, Chinese Academy of Sciences). All oligonucleotides and primers used in plasmid construction are listed in the supplemental tables. siRNA (siRPL39) or plasmids were transfected using Lipofectamine 2000 reagent (Invitrogen, U.S.) according to the manufacturer’s instructions. Retroviral production and infection were performed as previously described [16]. Stable cell lines were selected after treatment with 1 μg/ml puromycin (Sigma, U.S.) for 5 days. Surviving cells were trypsinized and diluted in an appropriate medium for colony formation analysis. Stable OVCAR3 and CAOV3 cell lines expressing AGK and AGK shRNA(s) were established (OVCAR3/CAOV3-AGK and OVCAR3/CAOV3-Vector, OVCAR3/CAOV3-shAGK-RNAi and OVCAR3/CAOV3-shAGK-Vector). Primers used in this study are presented in Supplemental Table 2.

### Western blotting

Western blotting was performed through the previously described methods [17] using an anti-AGK antibody (Abcam, U.S.) and antibodies against p27^Kip1^, p21^Cip1^, cyclin D1, phosphorylated-Rb as well as Rb (Cell Signaling, U.S.). The membranes were stripped and re-probed with an anti-GAPDH antibody or anti-α-tubulin antibody (Sigma, U.S.). The mitochondrial proteins were prepared using a cell mitochondria isolation kit (Abcam, U.S.). Antibodies used in this study are presented in Supplemental Table 3.

### Immunohistochemistry (IHC)

IHC was performed through the previously described methods [17]. The AGK, RPL39 and Ki67 primary antibodies used for IHC were respectively purchased from Abcam (U.S.), LSBio (U.S.) and Santa Cruz Biotechnology (U.S.). Mouse heart lysates were used as a positive control for AGK expression. The expression of RPL39 in hepatoma carcinoma cells and that of Ki67 in HeLa cells was served as a positive control. Antibodies used in this study are presented in Supplemental Table 3.

### Immunofluorescence (IF)

Cells were plated on coverslips (1 × 10^5^ cells) and cultured for 24 h, which were then fixed with 4% paraformaldehyde, permeabilized using 1% TritonX-100 and incubated with AGK as well as RPL39 antibodies at 4 °C overnight. Appropriate secondary antibodies were used to visualize AGK and RPL39. The nucleus was stained with DAPI (4′, 6-diamidino-2-phenylin-dole). Images were captured with an Olympus BX51 fluorescence microscope. Antibodies used in this study are presented in Supplemental Table 3.

### Immunoprecipitation

Mitochondria (0.5 mg of protein) were solubilized in 0.25 ml of digitonin-containing buffer (0.5% digitonin, 20 mM Tris-Cl, pH 7.4, 0.1 mM EDTA, 50 mM NaCl, 10% glycerol) with protease inhibitor (Roche, China) for 60 min at 4 °C. Solubilization buffer (digitonin-containing buffer without detergent) was added to the supernatant to achieve the final concentration of digitonin, which was 0.1% before being added to pre-equilibrated anti-FLAG affinity gel (Sigma-Aldrich, China). Next, washing was done in 0.1% digitonin-containing buffer, and bound protein complexes were eluted using 0.2 M glycine (pH 2.5).

### Mito-tracker and mitochondrial permeability transition pore (mPTP)

Cells were plated on coverslips (1 × 10^5^ cells) and cultured for 24 h. MitoTracker Red CMXRos (1 Mm) (Invitrogen, China) was pre-warmed at 37 °C before being added to the adherent cells. Cells were washed with pre-warmed cell medium, incubated at 37 °C for 30 min, then washed and fixed in 3.7% formaldehyde at 37 °C for 15 min, after which they were permeabilized in PBS containing 0.2% TritonX-100 for 10 min and incubated with AGK as well as RPL39 antibodies at 4 °C overnight. Appropriate secondary antibodies were used to visualize AGK and RPL39. The nucleus was stained with DAPI. The mPTP was determined through a mitochondrial membrane potential assay kit with JC-1 from Beyotime Biotechnology, China. Confocal laser scanning microscope imaging was carried out through an Olympus FV 3000 confocal microscopy.

### Cell growth curve, colony formation assay and EdU assay

The cells were plated in 6-well plates (1 × 10^5^ cells per well) and cultured for 5 days, which were counted every day to draw the cell growth curve and plated at 5 × 10^2^ cells/well in 6-well plates for colony formation assay. After 10 days, colonies were fixed with 4% aldehyde for 5 min and dyed with 1% crystal violet (Sigma, U.S.) for 30 s, which were then photographed and counted. The EdU assay was performed according to the manufacturer’s instructions (Rio-Bio, China). Typically, cells were counted in 10 fields of view through a microscope using × 200 magnification. Reagents used in this study are presented in Supplemental Table 4.

### Flow cytometry and Hoechst staining

Flow cytometry was performed using a BD FACS Aria II cell sorter (Becton Dickinson, San Jose, CA) to analyze cell cycle through propidium iodide (Sigma, China) staining. Modfit LT 3.1 trial cell cycle analysis software was used to analyze the cell cycle. The cells of Hoechst-stained-side population were resuspended at 10^6^ cells/ml in pre-warmed DMEM containing 2% FBS, penicillin, streptomycin, 1 mM HEPES and 5 g/ml Hoechst 33342 (Sigma, China) for 90 min at 37 °C. Laboratory apparatuses used in this study are presented in Supplemental Table 5.

### Sphere formation assays

The cells were grown in ultralow cluster attachment plates (Corning, U.S.) for tumor sphere formation assays. Spheres were cultured in a sphere medium, including DMEM/F12 serum-free medium supplemented with 2% B27 (Invitrogen, U.S.), 20 ng/ml basic fibroblast growth factors, 20 ng/ml epidermal growth factors (Pepro Tech, U.S.), 0.4% bovine serum albumin (Pythonbio, China) and 5 μg/ml insulin (Pythonbio, China). 5000 cells were seeded in 6-well ultra-low cluster plates. The spheres were counted on Day 3, 6, 9 and 12, respectively, and those cultured on Day 21 were used to enrich EOC CSCs. To analyze serial sphere formation, spheres were washed in PBS twice and dissociated into single cells through trypsinization. Reagents used in this study are presented in Supplemental Table 4.

### Tumor xenografts assay

All experimental procedures were approved by the Institutional Animal Care and Use Committee of Southern Medical University. Five- and six-week-old female non-obese diabetic mice with severe combined immune deficiency (NOD-SCID) were purchased from Beijing Vital River Laboratory Animal Technology Company (Beijing, China), which were randomly divided into 4 groups (4 different dosages of total cells, 1 × 10^5^, 1 × 10^4^, 1 × 10 ^3^, 0; *n* = 5 per group). Cells with matrigel (final concentration was 25%) were inoculated into the subcutaneous inguinal folds of NOD/SCID mice. Tumor volume was calculated based on the eq. V = (length × width 2)/2. On Day 31, mice were euthanized and the tumors were excised as well as weighed. Magnetic resonance imaging (MRI) analysis was conducted through the Biospec USR47/40 (Bruker Biospin MRI, U.S.). Reagents used in this study are presented in Supplemental Table 4.

### Biotin identification (BioID) assay

BioID was conducted as previously described [18]. Briefly, AGK was expressed with a mutant *E.coli* biotin ligase (BirA R118G, or BirA*), which was fused to a bait of interest, allowing for the local activation of biotin and the subsequent biotinylation of proteins in the bait vicinity. During the incubation of cells with 50 μM of biotin for 18–24 h, the reactive biotinoyl-AMP molecule diffused away from the BirA* moiety to react with amine groups on lysine residues in polypeptides nearby. The labeling radius of BirA* was estimated to be around 10 nm. Following a stringent cell lysis, a streptavidin matrix was used to isolate biotinylated proteins, which were used for subsequent mass spectrometry analyses.

### Chemoresistance model in vitro and in vivo

The cells were plated in 6-well plates (1 × 10^5^ cells) and cultured until their confluency was 90%. Through flow cytometry, the annexin V+/PI ¯ cells were analyzed after the indicated cells were treated with cisplatin (40 μm/ml, 80 μm/ml, 120 μm/ml) for a 24 h culture. The apoptotic rates of OVCAR3-AGK and OVCAR3-vector cells were analyzed, which were treated with different dosages of cisplatin (40 μm/ml, 80 μm/ml, 120 μm/ml, 160 μm/ml, 200 μm/ml). After 24 h of treatment, viable cells were counted to draw a cell growth curve.

Five- and six-old female non-obese diabetic-severe mice with combined immune deficiency (NOD-SCID) were purchased from Beijing Vital River Laboratory Animal Technology Company (Beijing, China). Stably-overexpressed AGK and vector cells (5 × 10^6^) were intraperitoneally injected into the NOD/SCID mice subcutaneously. Five mice were in one group. Three days later, cisplatin (5 mg/kg) was injected intraperitoneally into nude mice 3 times per week. On Day 35, mice were euthanized and the tumors as well as ascites were excised and weighed.

### Microarray analysis

The microarray data was analyzed by Aksomics (Shanghai, China). Six matched pairs of fresh tumor tissue specimens and ANT samples were obtained from patients with EOC after surgery. The information of patients is listed in Supplemental Table 1. The microarray data was evaluated by SHBIO Biotechnology Corporation (Shanghai, China). Corresponding microarray data was downloaded from The Cancer Genome Atlas (https://cancergenome.nih.gov/). An analysis of microarray data was performed with MeV 4.6 software (http://www.tm4.org/mev/). The prognostic value of AGK and RPL39 in ovarian cancer was assessed using described previously Kaplan-Meier plotter analysis [19].

### Measurement of ATP concentration and activity for mitochondrial complex I, III, IV

The cell supernatant was collected and the ATP concentration was detected using an ATP assay kit (Beyotime Biotechnology, China). The activity of mitochondrial complex I, III, IV and ATPase was determined using a mitochondrial complex respiratory chain activity assay kit (Comin Biotechnology, China). Data was analyzed according to the manufacturer’s instructions.

### Measurement of mitochondrial DNA (mtDNA) level and intracellular reactive oxygen species (ROS) production

The ROS levels were determined with a DCFDA/H2DCFDA-cellular ROS assay kit according to the manufacturer’s instructions (Abcam, U.S.). Flow cytometry was conducted on a BD FACS Aria II model cell sorter (Becton Dickinson, U.S.) through flow cytometry. The mtDNA levels were measured with the mtDNA monitoring primer set according to the manufacturer’s protocol (TaKaRa, Japan).

### Statistical analyses

A statistical analysis was performed using Prism 7 (GraphPad) and IBM SPSS software package version 20.0. Overall survival (OS) was defined as the time from surgery to death or to the time point of the last follow-up. Progression-free survival (PFS) was defined as the length of time from treatment to the onset of recurrence or progression (diagnosed through imaging or clinical assessment). Kaplan-Meier curves were plotted to assess the effect of AGK expression level on PFS and OS. The survival curves were compared during a log-rank test. The relationship between the expression of AGK and clinicopathological characteristics was analyzed through Pearson’s χ2 and Fisher’s exact tests. A multivariate Cox regression analysis was performed for all clinicopathological variables that were found to be significant through a univariate analysis. Data was represented as the means ± standard deviation. In all tests, a two-sided *P*-value of less than 0.05 was considered statistically significant.

## Results

### High expression of AGK correlated with the progression and poor prognosis of EOC

**Fig. 1:**
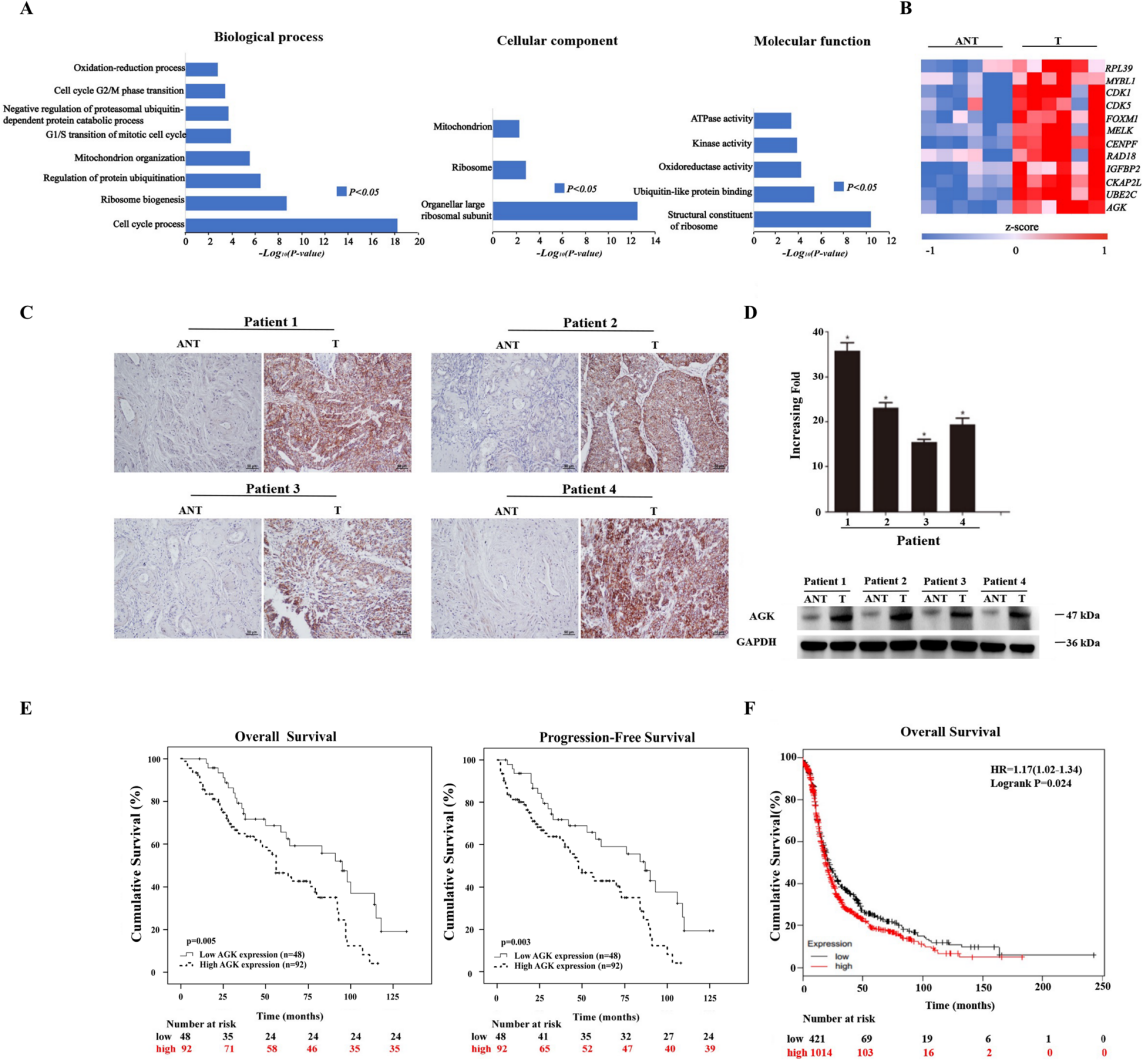
High expression of AGK correlated with EOC progression. **A** GO functional and pathway enrichment analysis of EOC specimens (T) compared to the adjacent noncancerous tissue samples (ANT). **B** A heatmap representing the genes upregulated in T compared to the adjacent ANT. 1 column represents 1 sample in different conditions. **C** Specimens derived from human EOC (T) were analyzed through IHC compared to the adjacent noncancerous tissue samples (ANT). AGK was upregulated in human EOC tissues. **D** RT-PCR and Western blotting were performed to analyze 4 matched pairs of EOC specimens (T) and adjacent noncancerous tissue samples (ANT). *N* = 3 independent experiments. **E** Kaplan-Meier overall survival curves (left panel), progression-free survival (right panel) and univariate analyses (log-rank) comparing patients with EOC with low (*n* = 48) and high (*n* = 92) AGK-expressing tumors. **F** The prognostic value of AGK in ovarian cancer series from publicly available datasets. Kaplan–Meier curves through a univariate analysis (log-rank) of patients with EOC and a high AGK expression (*n* = 1014) versus those with a low AGK expression (*n* = 421) for the overall survival (http://kmplot.com/analysis/index.php?p=service&cancer=ovar)

We analyzed the differences between gene expression profiles of 6 matched pairs of EOC and ANT samples through genome-wide cDNA array analysis. The result revealed that 12 genes, including AGK and RPL39, were significantly up-regulated in EOC tissues compared with adjacent noncancerous tissues (Fig. 1A and B). The clinical pathological features of patients with EOC for microarray analysis is presented in Supplemental Table 1. We further performed IHC, RT-PCR and Western blotting to analyze the AGK expression in other 4 matched pairs of EOC as well as ANT samples. The level of AGK mRNA and protein was upregulated in EOC tissues compared with the ANT (Fig. 1C, D).

Of the 140 patients with EOC, whose median age was 46 years (range 15 ~ 76 years), 30 were in Stage I, 23 were in Stage II, 77 were in Stage III and 10 were in Stage IV. All the 140 patients received initial treatments including surgeries and post-operational chemotherapies. Statistical analysis showed a significant correlation between the expression of AGK and the clinicopathological characteristics of EOC, including tumor stage (*P = 0.002*), peritoneal cytology (*P = 0.004*) and the volume of ascites (*P = 0.001*) (Table 1). Logistic multi-factor analysis revealed that the International Federation of Gynecology and Obstetrics (FIGO) stage and the expression of AGK were independent prognostic factors for patients with EOC (Table 2).

**Table 2:** Logistic multi-factors analysis the factors of up-regulated AGK expression in EOC patients

Variable	B	Wald	*P*	Exp(B)	95%CI
FIGO stage	0.989	0.049	**0.049**	2.688	0.861–8.391

***P < 0.05*** was considered statistically significant

We next performed a Kaplan-Meier analysis to investigate the relationship between the expression of AGK and the survival of patients with EOC, who were followed up for 1–133 months (median, 56 months), with 62 of them alive and 77 dead. The PFS of patients with a high and low/no expression of AGK was 42 months (range, 1–108 months) and 87 months (range, 5–127 months) respectively (log-rank test *χ*2 = 8.85, *P = 0.003*). The median OS of patients with a high and low/no expression of AGK was 54 months (range, 1–116 months) and 96 months (range, 11–133 months) respectively (log-rank test *χ*2 = 8.01, *P = 0.005*). These results revealed a clear negative correlation between the expression of AGK and PFS/OS of patients with EOC (Fig. 1E). We also determined the prognostic value of AGK in ovarian cancer using a Kaplan-Meier plotter. Clinical data is integrated with gene expression from 12 different datasets of 1648 patients in this database. We found that a high expression of AGK among 1014 patients with EOC was associated with a shorter OS compared with 412 of them with a low AGK expression (*HR = 1.17, Logrank P = 0.024*, Fig. 1F).

To determine whether AGK served as an independent prognostic factor, PFS and OS were assessed using a Cox proportional hazard model. Univariate analysis revealed that tumor stage, peritoneal cytology, the volume of ascites and the overexpression of AGK were associated with PFS and OS. Further analyses with a multivariate model showed that tumor stage (*P = 0.011*), optimal cytoreductive surgery (*P = 0.003*) and an upregulated AGK (*P = 0.036*) were independent prognostic factors for a poor PFS (Table 3). Similarly, Cox regression analysis revealed that tumor stage (*P = 0.008*), optimal cytoreductive surgery (*P = 0.001*) and an upregulated AGK (*P = 0.05*) were also independent prognostic factors for a poor OS. These findings further indicated that an upregulated AGK was associated with the aggressive progression and poor prognosis of patients with EOC.

**Table 3:** Multivariate analysis of prognostic factors for EOC patients (PFS & OS)

Outcomes	Variable	HR	P	95%CI
PFS	The volume of ascites	0.645	0.863	0.462–1.613
	Peritoneal cytology	1.535	0.135	0.875–2.694
	FIGO stage	2.359	***0.008***	1.255–4.435
	AGK expression	1.726	***0.050***	1.000–2.981
Outcomes	Variable	HR	P	95%CI
OS	The volume of ascites	0.864	0.649	0.461–1.619
	Peritoneal cytology	1.538	0.133	0.877–2.695
	FIGO stage	2.268	***0.011***	1.211–4.246
	AGK expression	1.802	***0.036***	1.038–3.127

*Abbreviations*: *LVSI* Lymphovascular space invasion, *CI* Confident interval, *HR* Hazard ratio, *OS* Overall survival, *PFS* Progression-free survival

***P < 0.05*** was considered statistically significant

### AGK promotes cell proliferation and regulates the G1-S phase transition

**Fig. 2:**
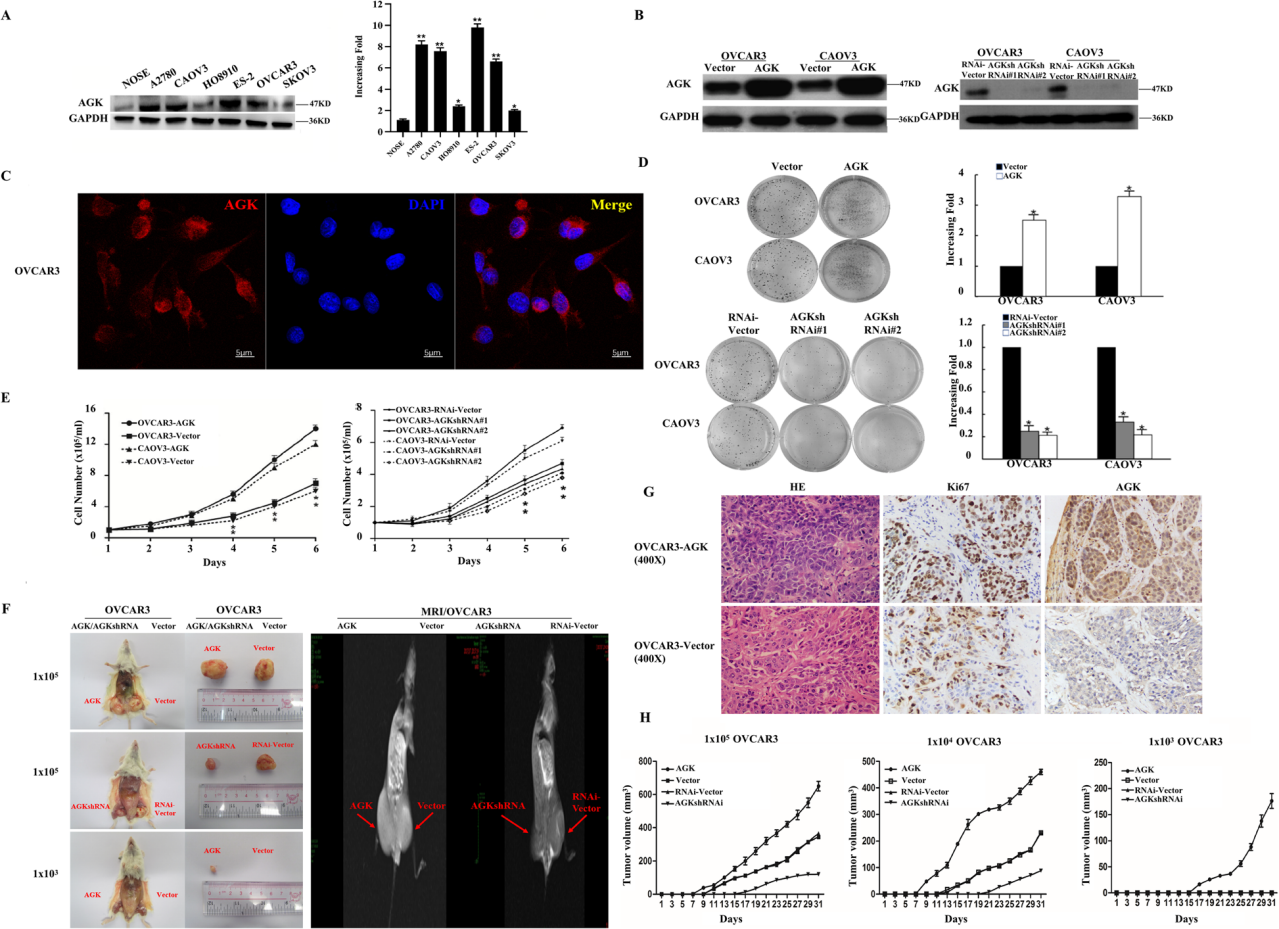
AGK promotes EOC cell proliferation in vitro and in vivo*.* **A** The level of AGK protein and mRNA in NOSE cells and 6 EOC cell lines was examined through Western blotting and quantitative real-time PCR (qPCR). GAPDH was used as an internal control. **B** Stably overexpressed and knocked-down AGK EOC cell lines (OVCAR3 and CAOV3) were detected by WB. **C** Immunofluorescence staining showed the expression of AGK (red) in EOC cells. Colony formation assay (**D**) and cell growth curve (**E**) indicated that the growth rate increased in cells with AGK overexpression and decreased in those with AGK knockdown. The number of cells on Day 2–6 was normalized to that of the cells on Day 1 as a control. The number of colonies was quantified through a colony formation assay. Each bar represents the mean ± SD of 3 independent experiments. *P < 0.05. **F** The generation of the xenograft model in NOD/SCID mice. OVCAR3-AGK, OVCAR3-AGK-RNAi and the respective control cells were inoculated into NOD/SCID mice (*n* = 5/group). Representative images of tumor-bearing mice (left panel) and the MRI images of tumors (right panel). **G** H&E staining of representative samples derived from mouse xenograft. The trend of the expression of AGK and Ki67 is consistent with that of staining using IHC. **H** The tumor volume of different groups was measured on the indicated days. Each bar represents the mean ± SD of 3 independent experiments. *P < 0.05. *N* = 3 independent experiments

We next performed RT-PCR and Western blotting analyses to determine the mRNA and protein level of AGK in 6 EOC cell lines as well as the immortalized primary NOSE cells. We found that the mRNA and protein level of AGK was higher in the 6 EOC cell lines than that in NOSE cells (Fig. 2A). To further investigate the potential biologic function of AGK, its stable overexpression and knockdown in EOC cells (OVCAR3, CAOV3) were established (Fig. 2B). AGK was mainly located in the cytoplasm (Fig. 2C). Colony formation assay and cell growth curve revealed that the overexpression of AGK significantly promoted cell proliferation compared with the control, whereas cell proliferation was significantly inhibited due to AGK knockdown (Fig. 2D, E). These results suggested that AGK played an important role in EOC cell proliferation in vitro.

We next explored the effect of AGK on EOC cells in vivo through a xenotransplantation experiment. Different numbers (1 × 10^5^, 1 × 10^4^, 1 × 10^3^) of overexpressed (OVCAR3-AGK) or knocked-down (OVCAR3-AGKshRNA) AGK cells mixed with 25% matrigel were collected and subcutaneously inoculated into the inguinal folds of NOD/SCID mice. As shown in Fig. 2F, the tumor size induced by the injection of OVCAR3-AGK cells and OVCAR3-AGK-RNAi was respectively significantly larger and smaller than OVCAR3-vector control cells. To further confirm these results, we performed additional in vivo MRI experiments on these tumor samples and found that tumor formation markedly increased with 1 × 10^3^ OVCAR3-AGK cells, presenting higher rates of tumorigenesis (Fig. 2H, Table 4). IHC results showed that the tumor areas with intense AGK staining revealed a high expression of Ki-67, whereas regions with weak AGK staining revealed a low expression of it. Image J was performed to analyze the AOD (average optical density) of IHC (Fig. 2G). These results demonstrated that AGK played a critical role in the tumorigenicity of EOC.

**Table 4:** Tumor numbers of different groups were measured on the indicated days

Inoculated Cells	Number of cells inoculated		
	1 × 10^5^	1 × 10^4^	1 × 10^3^
OVCAR3-Vector	5/5	3/5	0/5
OVCAR3-AGK	5/5	5/5	4/5
OVCAR3-RNAi-Vector	5/5	4/5	0/5
OVCAR3-AGK- RNAi	2/5	1/5	0/5

**Fig. 3:**
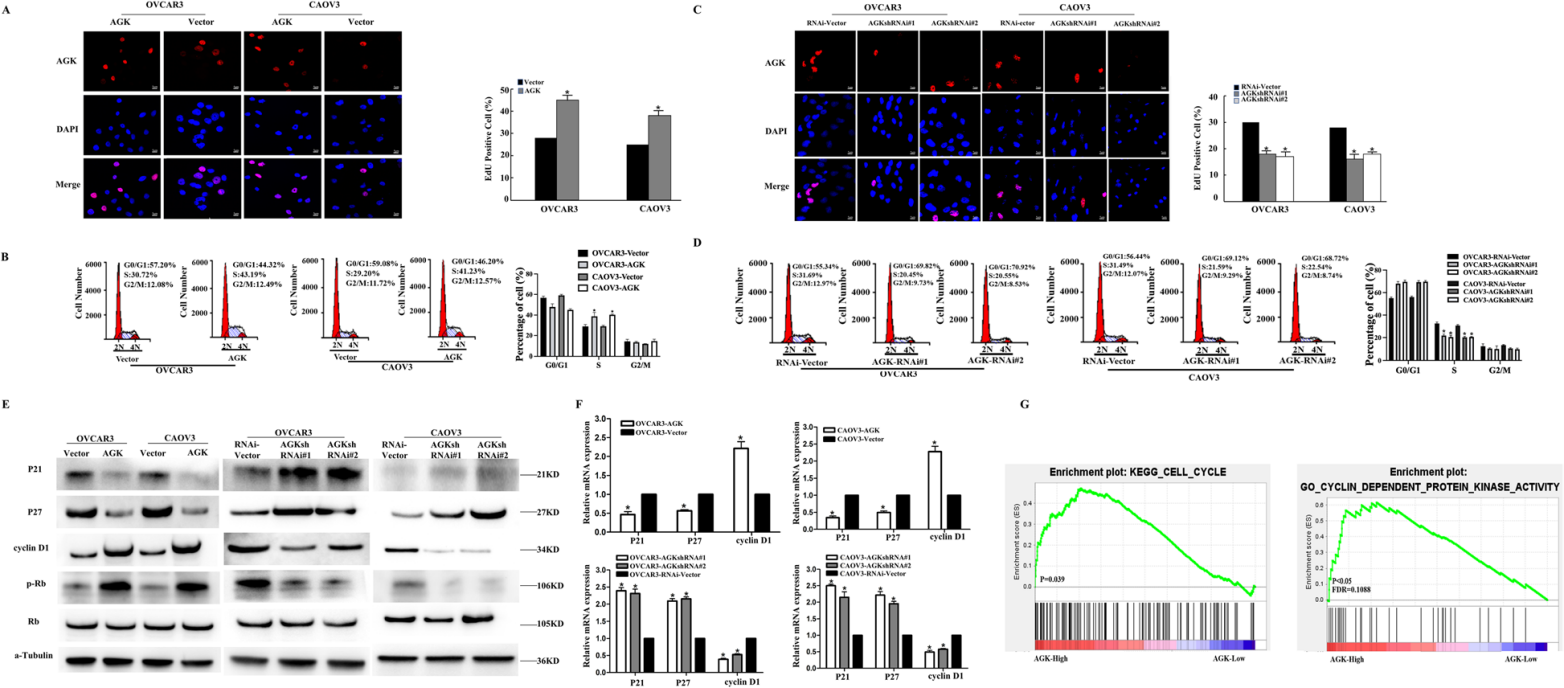
AGK regulates the G1-S-phase transition in EOC cells. **A** Representative micrographs (left panel) and the quantification (right panel) of EdU incorporation in the OVCAR3/CAOV3-AGK as well as vector control cells. **B** Cell cycle flow cytometric analysis of AGK-overexpressing and vector control cells. **C** Representative micrographs (left panel) and the quantification (right panel) of EdU incorporation in the OVCAR3/CAOV3-AGK-RNAi as well as vector control cells. **D** Cell cycle flow cytometric analysis of OVCAR3/CAOV3-AGK-RNAi and vector control cells. AGK altered the expression of G1-S-phase cell-cycle regulators. Western blotting (**E**) and real-time PCR (**F**) analysis of the expression of p21^Cip1^, p27^Kip1^, cyclin D1, p-Rb and the total Rb protein in the OVCAR3/CAOV3-AGK as well as OVCAR3/CAOV3-AGK-RNAi cells; α-Tubulin was used as a loading control. Each bar represents the mean ± SD of 3 independent experiments. *P < 0.05. *N* = 3 independent experiments. **G** GSEA plot analysis showed that the expression of AGK was positively correlated with cell cycle and cell-cycle-dependent protein kinase based on the published gene expression profiles of patients with EOC (the Cancer Genome Atlas TCGA, https://cancergenome.nih.gov/, GSE102180). All experiments were repeated at least three times independently

To further investigate the mechanisms through which AGK promoted the proliferation of EOC cells, we performed EdU and flow-cytometry assays on overexpressed and knocked-down AGK cells. As is shown in Fig. 3A-D, the proportion of S-phase cells significantly increased with the overexpression of AGK while contradictory results were got for AGK knockdown. We then analyzed the expression of p21^Cip1^ and p27^Kip1^ as well as the inhibitors of cyclin-dependent kinases (CDK) at both protein and mRNA level through Western blotting and real-time RT-PCR. Compared to the control cells, CDK was drastically reduced in overexpressed AGK cells (Fig. 3E and F). This was accompanied by a simultaneous increased number of cell cycle regulators p-Rb and cyclin D1 (Fig. 3E and F). An opposite result was obtained from the knocked-down AGK cells. We therefore demonstrated that AGK expedited the G1-S transition and promoted the proliferation of EOC. Consistent with the above results, gene set enrichment analysis (GSE102180) and plot analysis showed that AGK expression between the normal and tumor tissues was positively correlated with cell cycle and CDK protein in gene expression profiles of patients with EOC from the TCGA database (Fig. 3G).

### AGK increases the stem cell population and promotes stem cell-like phenotype

**Fig. 4:**
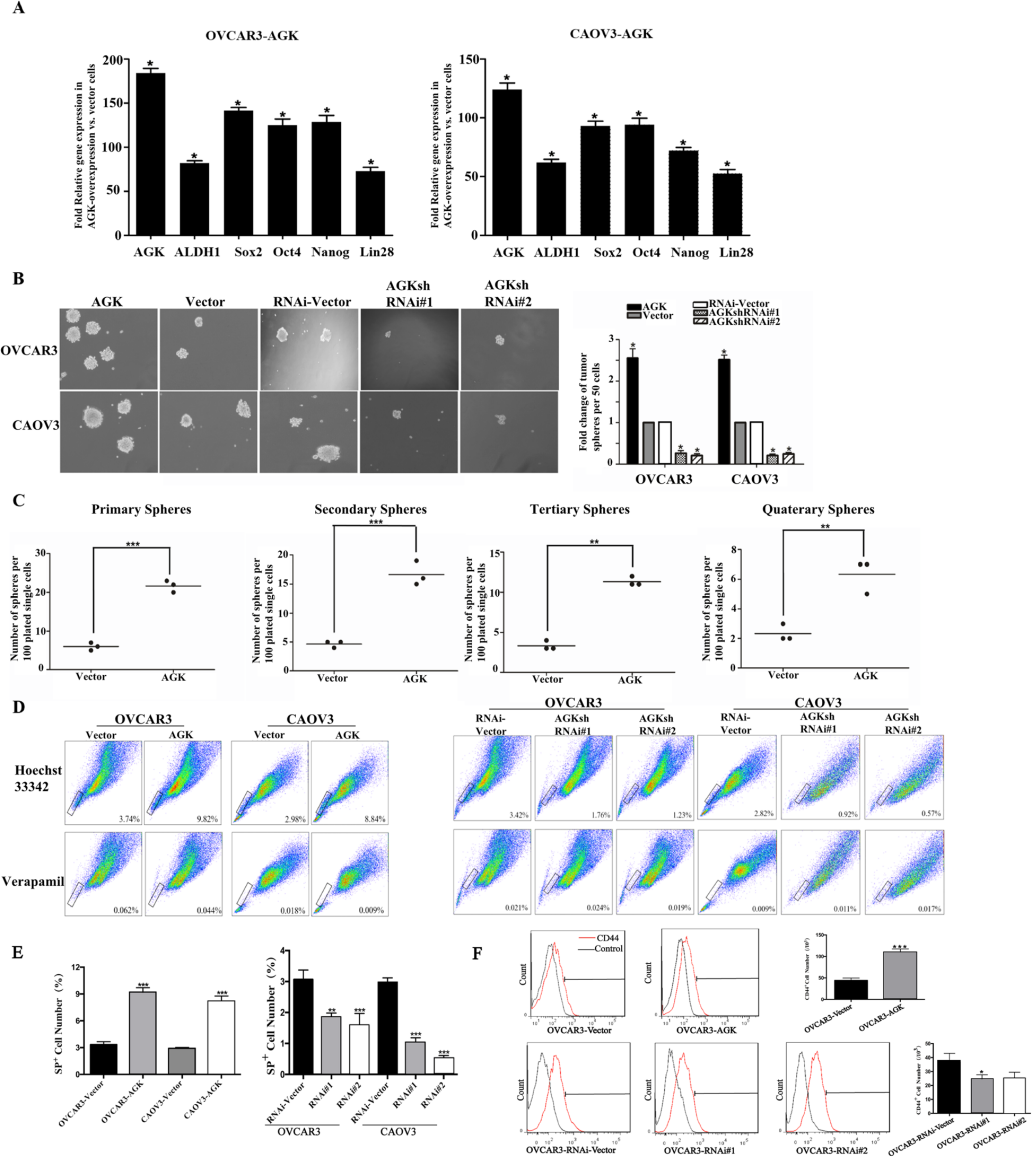
AGK increases the population of EOC CSC and induces chemoresistant in vitro. **A** A real-time PCR analysis of the expression of pluripotency-associated markers, ALDH1, Sox2, Oct4, Nanog and Lin28 in the indicated cells. **B** Representative images (left panel) of spheres formed by the indicated cells. Histograms (right panel) showed the mean number of spheres formed by the indicated cells. **C** Single-AGK-overexpressing and control cells were performed for their sphere-forming potential in 96-well plates. Tertiary and quaternary spheres were generated in cells dissociated from 1 previous individual generation sphere in each 96-well plate. Data was compiled from 3 independent biologic experiments. **D** Hoechst-stained side population (SP) assay showed that the overexpression of AGK was promoted, whereas AGK knockdown attenuated the SP cells in the indicated cells. **E** Histograms show the mean percent of SP^+^ cell numbers. **F** Flow cytometry analysis of CD44^+^ cells (left panel). Histograms (right panel) show the mean percent of CD44+ cells

Since AGK promoted tumorigenicity of EOC cells, we hypothesized that AGK might enrich the CSC population in EOC. The results showed that the overexpression of AGK in both OVCAR3 and CAOV3 cells increased in the number of EOC stem cell markers, ALDH1, SOX2, Oct4, Nanog and Lin28 (Fig. 4A), indicating that the overexpression of AGK enhanced stem cell-like phenotypes in EOC.

We next assessed the effect of AGK expression on sphere formation, an index of self-renewal ability, using tumor sphere formation assay (Fig. 4B). The result revealed that overexpressed AGK cells formed more than 2.5-fold larger spheres than the controls, while knocked-down AGK cells formed less than 4-fold smaller spheres than the controls. Single-cell tumor sphere assays confirmed the higher frequency of sphere-initiating cells in overexpressed AGK cells (Fig. 4C). SP^+^ and CD44^+^ have recently been reported as potential CSC markers of the majority of cancers [20]. We then further examined the effect of AGK on the regulation of the proportion of SP + cells and CD44 + cells. As is shown in Fig. 4D and E, with the overexpression of AGK, the proportion of SP^+^ cells increased from 3.74 to 9.82% in OVCAR3 cells and from 2.98 to 8.84% in CAOV3 cells respectively. Conversely, with knocked-down AGK cells, the proportion of SP^+^ cells decreased from 3.42 to 1.76% in OVCAR3 cells and from 2.82 to 0.92% in CAOV3 cells respectively. Similarly, the CD44^+^ population dramatically increased in overexpressed AGK cells but decreased in knocked-down AGK cells (Fig. 4F). These results demonstrated that AGK promoted the stem cell-like phenotypes and increased the stem cell population in EOC.

### AGK enhances apoptosis resistance

**Fig. 5:**
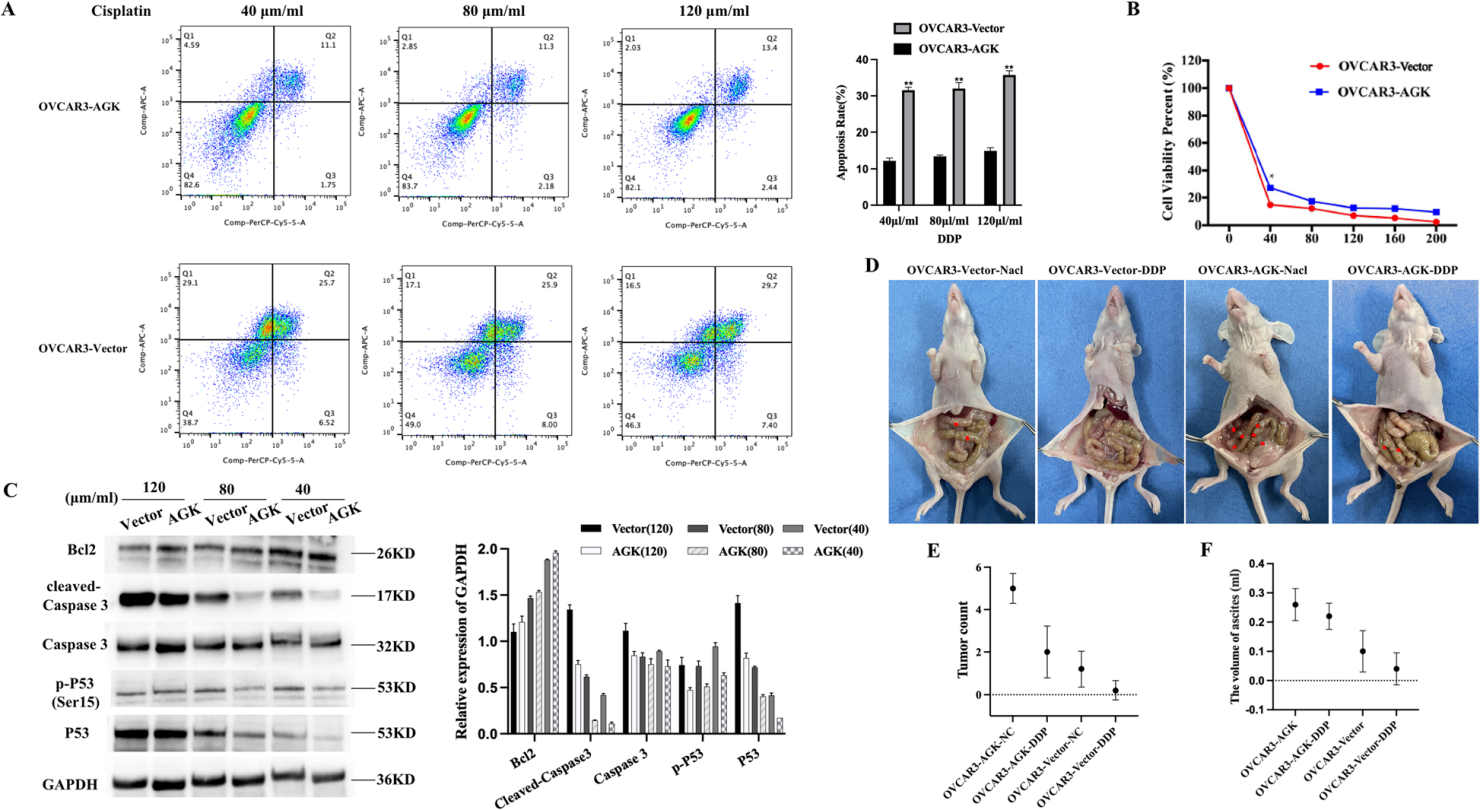
AGK increases the population of EOC CSC and induces chemoresistant in vivo. A Flow cytometry analysis of annexin V^+^/PI ¯ cells after the indicated cells were treated with cisplatin (40 μm/ml, 80 μm/ml, 120 μm/ml) for 24 h. Results were expressed as percentages of total cells. **B** The dose-dependent curve of cisplatin treatment for OVCAR3-AGK and OVCAR3-Vector cells. **C** Cleaved caspase 3, caspase 3, Bcl-2, p-P53, and P53 were apoptosis markers tested by WB. **D** Cisplatin chemoresistance of AGK on EOC in vivo. AGK-overexpressing (OVCAR3-AGK) or AGK-knockdown (OVCAR3-AGKshRNA) cells (5 × 10^6^) were injected subcutaneously into the subcutaneous and intraperitoneal inguinal folds of NOD/SCID mice. 3 days later, cisplatin (5 mg/kg) was injected intraperitoneally into nude mice 3 times per week. On Day 25, mice were euthanized and the tumors as well as ascites were excised and weighed. A chemiluminescent imaging system (Sacecreation) was used to evaluate the tumors in the mice. Error bars represent the means ± SD of 3 independent experiments. *P < 0.05. **P < 0.001. *N* = 3 independent experiments

Cisplatin is one of the most widely used and highly effective drugs for the treatment of various solid tumors. However, cisplatin chemoresistance is a clinical problem that leads to treatment failure of EOC [21, 22]. To investigate the role of chemotherapeutic sensitization in EOC cells, OVCAR3-vector and OVCAR3-AGK cells were stained with annexin V and PI. The results revealed that the overexpression of AGK induced apoptosis resistance of cisplatin-treated EOC cells (Fig. 5A). Furthermore, we measured the number of viable cells in OVCAR3-vector and OVCAR3-AGK cells in response to cisplatin treatment. Our findings showed that the cell survival increased and the sensitivity of EOC cells decreased to cisplatin in a dose-dependent manner with the overexpression of AGK (Fig. 5B). Cleaved caspase 3, caspase 3, Bcl-2, p-P53 and P53 were apoptosis markers tested by WB. The results revealed that AGK overexpression enhanced apoptosis resistance in cisplatin-treated EOC cells (Fig. 5C).

We next explored the cisplatin chemoresistance of AGK on EOC cells in vivo through a xenotransplantation experiment. Overexpressed (OVCAR3-AGK) AGK cells (5 × 10^6^) were injected subcutaneously into the intraperitoneal inguinal folds of NOD/SCID mice. Three days later, cisplatin (5 mg/kg) was injected intraperitoneally into nude mice 3 times per week. As shown in Fig. 5D-F, OVCAR3-AGK cells injection produced more tumors and ascites than vector cells. Six total injections of cisplatin (5 mg/kg) were given, and neither the tumor counts nor the ascites volume in the OVCAR3-AGK cells group significantly decreased. The findings showed that cisplatin resistance in EOC was enhanced by AGK.

### AGK maintains mitochondrial structure and function

**Fig. 6:**
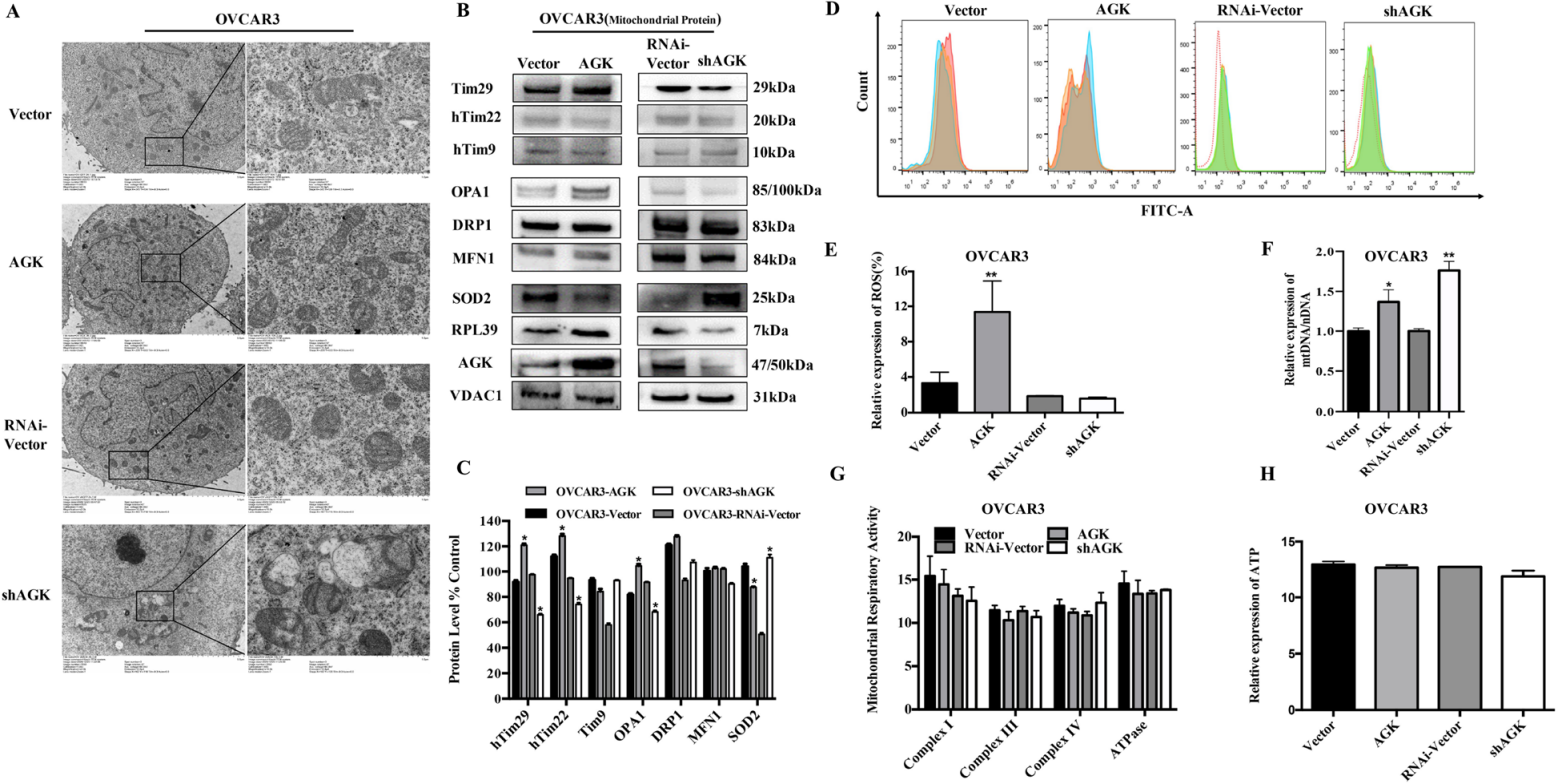
AGK maintains the mitochondrial morphology and function of EOC cells. **A** Transmission electron microscopic analysis of OVCAR3-AGK and OVCAR3-shAGK cells. Scale bar was 1 μm. **B** Western blotting analysis of indicated cell lines using antibodies against Tim29, hTim22, hTim9, OPA1, DRP1, MFN1, SOD2, RPL39, AGK and VDAC1. (**C**) Analysis of differential expressions of Tim29, Tim22, Tim9, OPA1, DRP1, MFN1 and SOD2 in Image J. Error bars represent the means ± SD of 3 independent experiments. *P < 0.05. **D**, **E** Flow cytometer analysis of the cellular ROS production of OVCAR3-AGK and OVCAR3-shAGK cells through DCFDA/H2DCDA-cellular ROS Assay. Error bars represent the means ± SD of 3 independent experiments. *P < 0.05. **F** mtDNA (**G**) Mitochondrial respiration and (**H**) ATP were analyzed using a mtDNA monitoring primer set, a mitochondrial respiration assay kit and an ATP assay kit respectively. *N* = 3 independent experiments

AGK is a subunit of the TIM22 complex in the inner membrane of mitochondria. Therefore, we used transmission electron microscopy to assess the mitochondrial morphology of overexpressed (OVCAR3-AGK) and knocked-down (OVCAR3-AGKshRNA) AGK cells. A significantly decreased number of cristae in mitochondria of OVCAR3-AGKshRNA was observed compared to those in OVCAR3-AGK cells (Fig. 6A). Next, the mitochondria were isolated from OVCAR3-AGK and OVCAR3-AGKshRNA cells. The expression of mitochondrial fusion and fission-related genes was measured. MFN1 and DRP1 were expressed at similar levels in OVCAR3-AGK and OVCAR3-AGKshRNA cells. However, the expression of OPA1 increased in OVCAR3-AGK cells and decreased in OVCAR3-AGKshRNA cells (Fig. 6B and C). Since the biological function of OPA1 is to regulate mitochondrial fusion and maintain cristae structure [23], our results suggested that AGK maintained the structural integrity of mitochondria in EOC cells via an upregulated expression of OPA1.

Previous studies showed that the level of hTim22 and Tim29 reduced with AGK knockdown, resulting in an irreversible destruction of TIM22 complex substrates [24]. We measured the level of ROS production and examined the mtDNA damage in OVCAR3-AGK and OVCAR3-AGKshRNA cells. The results showed that cellular ROS production and Δψm increased. The expression of mitochondrial superoxide dismutase (SOD2) was suppressed in OVCAR3-AGK cells (Fig. 6D, E and Supplemental Fig. 1). mtDNA damage was lower in OVCAR3-AGKshRNA cells (Fig. 6F). To examine whether AGK affected other mitochondrial functions, we examined its role in mitochondrial respiration and measured the level of ATP (Fig. 6G and H), but there was no difference between OVCAR3-AGK, OVCAR3-AGKshRNA cells and control cells.

### RPL39 is involved in AGK pathway by directly interacting with AGK

**Fig. 7:**
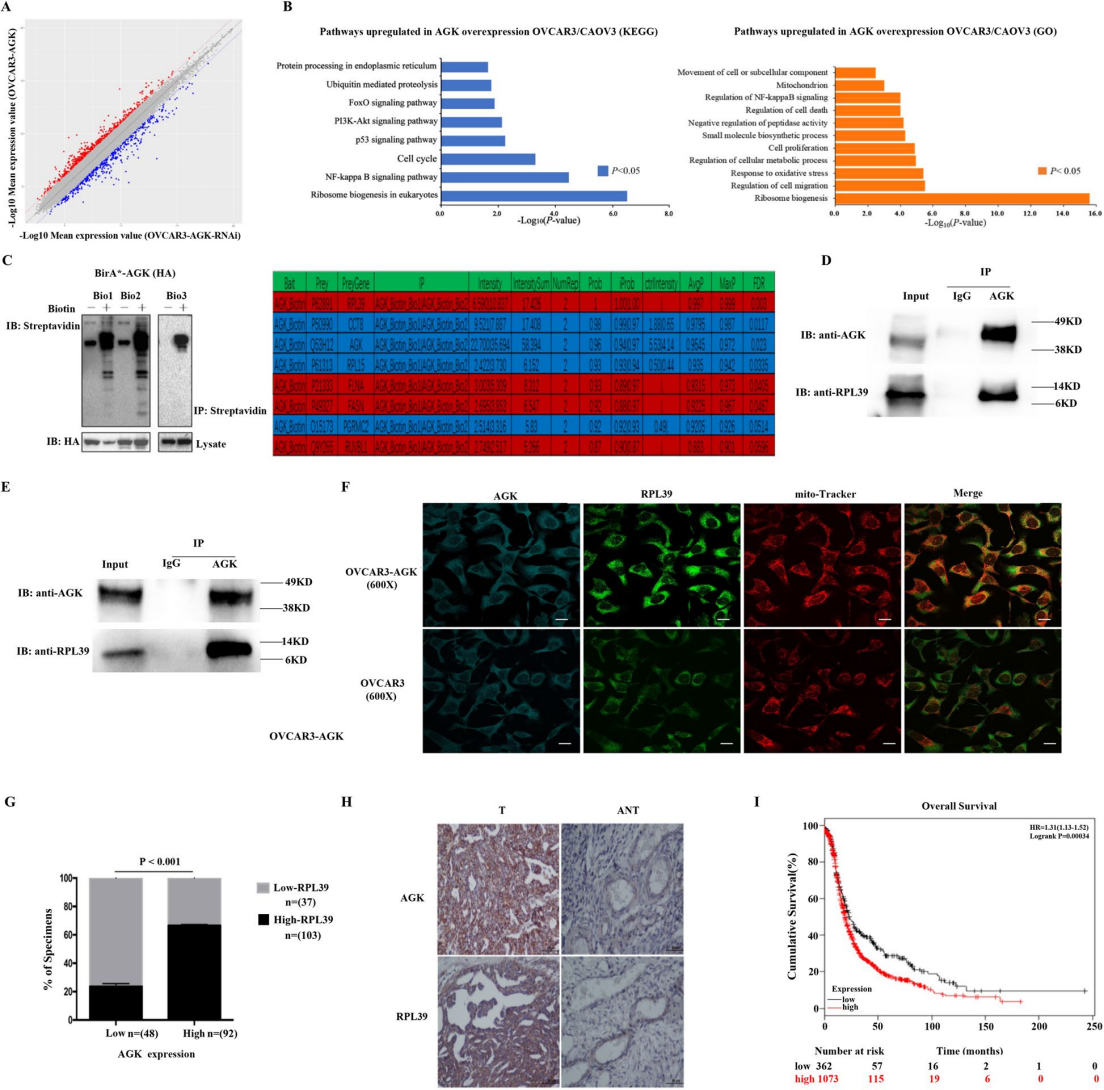
AGK directly interacts with RPL39. **A** Scatter plot comparing with global gene-expression profiles of OVCAR3-AGK and OVCAR3-AGK-RNAi cells. **B** A functional enrichment analysis of KEGG pathways and GO upregulated in OVCAR3-AGK cells compared to OVCAR3-AGK-RNAi. **C** Streptavidin immunoblot analysis (left panel). A mass spectrometry analysis of Bio-ID assay (right panel). **D** Co-immunoprecipitation assay revealed that AGK interacted with RPL39 (total protein). **E** Co-immunoprecipitation assay revealed that AGK interacted with RPL39 in mitochondria (mitochondria protein). **F** Confocal images for AGK and RPL39 in mitochondria through Mito-Tracker. **G**, **H** The correlation between the expression of AGK and RPL39 in patients with EOC through IHC. The expression of AGK was positively correlated with the expression level of RPL39, *P* < 0.001. **I** The prognostic value of RPL39 in ovarian cancer series from the same publicly available datasets (http://kmplot.com/analysis/index.php?p= service&cancer = ovar). Kaplan–Meier curves through a univariate analysis (log-rank) of patients with EOC and a high expression of RPL39 (*n* = 1073) versus those with a low expression of RPL39 (*n* = 362) for the overall survival

We performed a microarray analysis to compare the gene expression profile of EOC cells with AGK overexpression and knockdown (OVCAR3, CAOV3). We also used the BioID method to identify the factors interacting with AGK in OVCAR3-AGK cells. As is shown in Fig. 7A, the KEGG analysis revealed that AGK significantly activated OVCAR3/CAOV3 ribosome biogenesis in cell eukaryotes. The KEGG and GO analysis demonstrated that AGK prominently activated cell ribosome biogenesis (Fig. 7B). Using the BioID method, we found that RPL39 interacted with AGK, which was further confirmed through co-immunoprecipitation (Fig. 7C and D).

Previous studies have shown that the function of RPL39 was to regulate ROS metabolism [25]. We were interested in the relationship between RPL39 and AGK in mitochondria, and found that RPL39 was expressed (Fig. 6B) as well as located (Fig. 7F) in the mitochondria. We further demonstrated that AGK and RPL39 physically interacted through co-immunoprecipitation experiments with purified isolated mitochondria protein (Fig. 7E). We then examined the relationship between AGK and RPL39 in patients with EOC. The tumor tissues with a high level of AGK staining revealed strong RPL39 signals. In contrast, those with a low level of AGK staining showed a weak RPL39 expression (*P* < 0.001), suggesting that the expression of AGK was positively correlated with that of RPL39 (Fig. 7G and H). We analyzed the prognostic value of RPL39 in patients with ovarian cancer, and found that the overexpression of RPL39 protein in 1074 patients with EOC was associated with a shorter OS than that in the 361 of those with a lower AGK protein expression (*HR = 1.31, Logrank P = 0.00034*, Fig. 7I).

### RPL39 cooperates with AGK in function

**Fig. 8:**
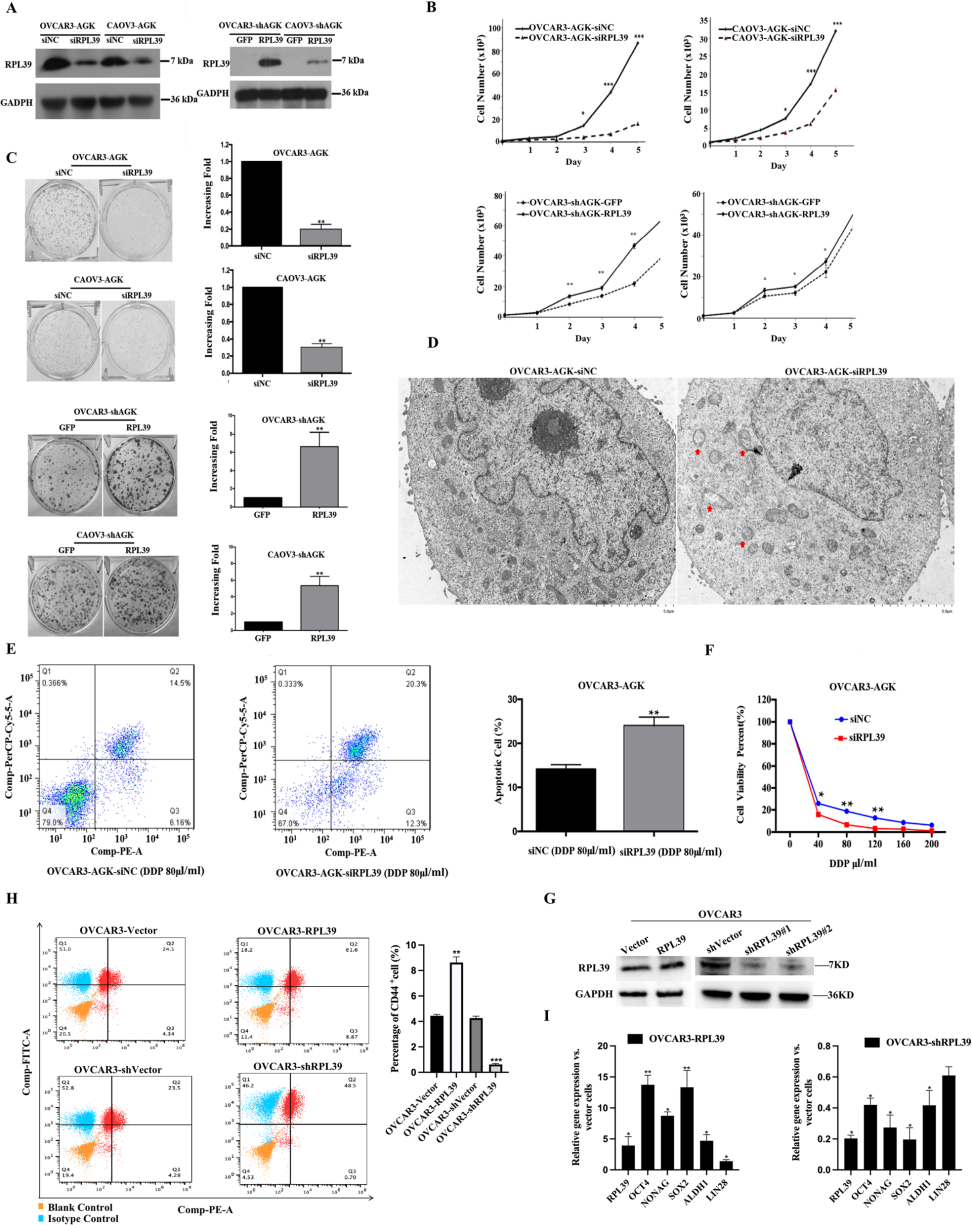
RPL39 cooperates with AGK in function. **A** The silencing of RPL39 in OVCAR3-AGK cells was determined through Western blotting and real-time PCR. The overexpression of RPL39 in OVCAR3-AGK-RNAi cells was confirmed through Western blotting. Cell growth curve (**B**) and colony formation assay (**C**) indicated that the growth rate decreased in OVCAR3-AGK-siRPL39 cells and increased in OVCAR3-shAGK-siRPL39 cells. The number of cells on Day 2–6 was normalized to those on Day 1 as a control. The number of colonies was quantified through the colony formation assay. **D** Transmission electron microscopic analysis of OVCAR3-AGK-siNC and OVCAR3-AGK-siRPL39 cells. Scale bar was 1 μm. **G** Stably overexpressed and knocked-down RPL39 EOC cell lines (OVCAR3) were detected by WB. **H** Flow cytometry analysis of annexin V^+^/PI ¯ cells after the indicated cells were treated with cisplatin (80 μm/ml) for 24 h. Results were expressed as a percentage of total cells. **I** The dose-dependent curve of cisplatin treatment for OVCAR3-AGK-siNC and OVCAR3-AGK-siRPL39 cells. Error bars represent the means ± SD of 3 independent experiments. *P < 0.05. **P < 0.001. *N* = 3 independent experiments

To further investigate the potential biological function of RPL39, the effect of silenced and overexpressed RPL39 in OVCAR3/CAOV3-AGK as well as OVCAR3/CAOV3-AGKshRNA cells was analyzed (Fig. 8A). The results of the cell growth curve and colony formation assays revealed that the OVCAR3/CAOV3-AGK-siRPL39 cells had a significantly lower proliferation rate, while the OVCAR3/CAOV3-AGKshRNA-RPL39 cells had a significantly higher proliferation rate compared to controls (Fig. 8B and C).

The mitochondrial morphology of OVCAR3-AGK-siNC and OVCAR3-AGK-siRPL39 cells was observed through transmission electron microscopy. The number of mitochondrial cristae in OVCAR3-AGK-siRPL39 cells significantly decreased compared to that in OVCAR3-AGK-siNC cells (Fig. 8D). To examine the effect of RPL39 on the sensitivity of OVCAR3-AGK cells in chemotherapeutics, we stained OVCAR3-AGK-siNC and OVCAR3-AGK-siRPL39 cells with annexin V and PI. Furthermore, the viable OVCAR3-AGK-siNC and OVCAR3-AGK-siRPL39 cells treated with increasing doses of cisplatin were counted. The cisplatin resistance of OVCAR3 cells induced by the overexpression of AGK attenuated the expression of siRNA-expressing lentiviral-vector-targeting RPL39 (Fig. 8E and F). Stable overexpression and knockdown of RPL39 in OVCAR3 were established (OVCAR3-RPL39, OVCAR3-Vector/OVCAR3-shRPL39, and OVCAR3-shVector) (Fig. 8G). EOC stem cell markers, ALDH1, SOX2, Oct4, Nanog, and Lin28 were increased in OVCAR3-RPL39 and decreased in OVCAR3-shRPL39. Similarly, the CD44+ population dramatically increased in OVCAR3-RPL39 cells but decreased in RPL39 knockdown cells as shown in Fig. 8H and I**.** This result clearly indicate that RPL39 is involved in AGK pathway by directly interacting with AGK.

## Discussion

AGK is a recently discovered mitochondrial lipid kinase [26]. Several previous studies provided evidences that the expression of AGK is upregulated in breast cancer, kidney cancer and nasopharyngeal cancer [27, 28]. However, its specific role in EOC remains unclear. In the present study, we found that AGK was upregulated in human EOC tissues, whose expression was significantly related to the poor clinicopathologic characteristics of EOC, including tumor stage, peritoneal cytology and the volume of ascites. The survival analyses revealed that patients with EOC and a higher AGK expression had a shorter OS and PFS than those with a lower AGK expression. We further found that the tumorigenicity of EOC cells increased with an upregulated AGK. In vivo experiments demonstrated that the xenotransplantation of AGK-overexpressing EOC cells resulted in a high level of tumorigenesis among NOD/SCID mice. These results provided strong evidence that AGK was an important oncogenic factor in the progression of EOC.

We here showed that AGK promoted the progression of EOC cell cycle from Stage G0/G1 into S and Stage G2/M by decreasing the activity of CDK inhibitors p21^Cip1^ and p27^Kip1^ as well as enhancing the activity of cyclin D1. This result was consistent with that of previous reports that the expression level of p21^Cip1^ and p27^Kip1^ was reduced by AGK, and the expression of cyclin D1 increased to mediate proliferation in breast cancer [29]. GSEA plot analysis also showed that the expression of AGK was positively correlated with the EOC cell cycle. A possible mechanism is that p21^Cip1^ and p27^Kip1^ abolished cyclin D1 nucleocytoplasmic shuttling, a process involved in promoting abnormal cell survival, tumor progression and drug resistance [30]. Therefore, our study demonstrated that AGK promoted EOC proliferation and survival by disrupting the normal cellular processes.

In the present study, we found that AGK enhanced the tumor sphere potential and the expression of stemness genes increased, such as ALDH1, SOX2, OCT4, NANOG, LIN28 and EOC stemness gene CD44. Also, with AGK, the proportion of SP^+^ increased and apoptosis resistance was enhanced in EOC cells. We further found that the sensitivity of EOC cells decreased to cisplatin in a dose-dependent manner due to the overexpression of AGK, suggesting that AGK was involved in the chemotherapy resistance of EOC in vitro and in vivo. Based on the above research evidence, we speculated that AGK might serve as a new biomarker and target in EOC diagnosis and therapies. Many previous studies have shown that identifying the CSC-specific markers provided a novel opportunity for treating cancers [31, 32]. Targeted strategies for the destruction of CSCs can also be used to significantly improve chemotherapeutic efficiency [33]. Interestingly, we found that the percentage of sphere-initiating cells increased with AGK, but primary-tumor spheres were only initiated in a subset of AGK-overexpressing cells. It appeared that AGK-induced CSCs only occurred in a subgroup of EOC.

In this study, we found that the number of mitochondria cristae was significantly reduced due to AGK knockdown, suggesting that the biological function of AGK was associated with mitochondria oxidative metabolism. Furthermore, we demonstrated a physical interaction between AGK and RPL39 in mitochondria. The functional interaction between AGK and RPL39 in mitochondria was required to maintain the mitochondrial structure and function. We also found that RPL39 promoted the activity of AGK, and AGK-promoted chemoresistance was enhanced by the expression of RPL39 through increasing the self-renewal ability of EOC CSCs. These results revealed that AGK induced EOC CSCs and drove chemoresistance by directly interacting with RPL39.

RPL39 is a component of large 60S ribosomal protein subunits regulating the activators of mitochondrial biogenesis. Recent studies revealed that RPL39 was involved in the development of several types of cancers. The expression of RPL39 was found to impact the capacity of self-renewal and drug resistance of breast CSC in lung cancer [34, 35]. Depletion of RPL39 suppressed the proliferation of pancreatic cancer [36]. However, the mechanism of RPL39 involved in cancer progression remains largely unclear. Our study provided important experimental evidence that RPL39 was an EOC-CSCs-related gene involved in cancer progression, chemoresistance and CSC formation by directly interacting with AGK in mitochondria.

## Conclusion

In summary, our study reveals that AGK is an EOC-related gene associated with the progression and poor prognosis of EOC. Furthermore, we demonstrate that the properties of human EOC CSCs increase with AGK, which induces chemoresistance by interacting with RPL39 in mitochondria. This functional interaction between AGK and RPL39 is necessary for maintaining mitochondrial structure and function. Therefore, targeting AGK to induce apoptotic responses of EOC CSCs is a novel strategy to treat patients with EOC.

## Supplementary Information

**Additional file 1: Supplemental Table 1.** The clinical pathological features of EOC patients for microarray (*n*=6). **Supplemental Table 2.** Primers used in this study. **Supplemental Table 3.** Antibodies used in this study. **Supplemental Table 4.** Reagents used in this study. **Supplemental Table 5.** Laboratory apparatus used in this study.

**Additional file 2: Supplemental Figure 1.** AGK enhances the level of Δψm in OVCAR3 cells.

## Data Availability

All data supporting this study are available within this article and supplementary Information file. The dataset used and/or analyzed during the current study are available from the corresponding author on reasonable request.

## Abbreviations

EOC: Epithelial ovarian cancer
CSCs: Cancer stem cells
AGK: Acylglycerol kinase
RPL39: Ribosomal protein L39
ANT: Adjacent noncancerous tissue
IHC: Immunohistochemistry
FBS: Fetal bovine serum
DMEM: Dulbecco’s modified Eagle medium
OVCAR3: Ovarian cancer cell line
NOSE: Normal ovarian surface epithelial
IF: Immunofluorescence
DAPI: 4′, 6-diamidino-2-phenylin-dole
mPTP: Mitochondrial permeability transition pore
NOD-SCID: Non-obese diabetic-severe combined immune deficiency
MRI: Magnetic resonance imaging
BioID: Biotin identification
mtDNA: Human mitochondrial DNA
ROS: Reactive oxygen species
OS: Overall survival
PFS: Progression-free survival
FIGO: International Federation of Gynecology and Obstetrics
CDK: Cyclin-dependent kinases
GSEA: Gene set enrichment analysis
SOD2: Mitochondrial superoxide dismutase
