# Supplementary material for: Acylglycerol kinase promotes ovarian cancer progression and regulates mitochondria function by interacting with ribosomal protein L39
Source: J Exp Clin Cancer Res. 2022 Aug 8;41:238. doi: 10.1186/s13046-022-02448-5 (PMC9358817; doi:10.1186/s13046-022-02448-5)
Supplement: Supplementary file 1 — Additional file 1: Supplemental Table 1. The clinical pathological features of EOC patients for microarray (n=6). Supplemental Table 2. Primers used in this study. Supplemental Table 3. Antibodies used in this study. Supplemental Table 4. Reagents used in this study. Supplemental Table 5. Laboratory apparatus used in this study. [file 13046_2022_2448_MOESM1_ESM.docx]

**Supplemental Table 1. The clinical pathological features of EOC patients for microarray (n=6)**

| **Medical record No.** | **Sample ID** | **Age(years)** | **Pathological type** | **FIGO stage** | **pathology grade** | **Postoperative treatment** |
| --- | --- | --- | --- | --- | --- | --- |
| 0000210074 | 1702 | 47 | Serous | IC | G1 | No |
| 0000210524 | 1711 | 59 | Serous | IIA | G1 | No |
| 0000210431 | 1708 | 53 | Serous | IIA | G2 | No |
| 0000210571 | 1709 | 66 | Serous | IIB | G2 | No |
| 0000296713 | 296713 | 44 | Mucinous | IIB | G3 | No |
| 0000294836 | 294836 | 55 | Mucinous | IIIA | G3 | No |

**Supplemental Table 2. Primers used in this study.**

| **ShRNA-AGK** | **Sequences** | **Companies** |
| --- | --- | --- |
| AGK-shRNAi#1: | GGAGAGACCAGTAGTTTGA | GENECHEM Shanghai |
| AGK-shRNAi#2: | GAGGCTACCTTCAGTAAGA | GENECHEM Shanghai |
| **Oligonucleotides** |  |  |
| GAPDH-F: | 5’ GCATCCTGGGCTACACTGAG 3’ | IGE GuangZhou |
| GAPDH-R: | 5’ CCACCACCCTGTTGCTGTAG 3’ | IGE GuangZhou |
| AGK-F: | 5’ GCCTCAGACTCATCAAGCCT 3’ | IGE GuangZhou |
| AGK-R: | 5’ GCTGCACATCTTTCCAGACC 3’ | IGE GuangZhou |
| OCT4-F: | 5’ GCCCGAAAGAGAAAGCGAAC 3’ | IGE GuangZhou |
| OCT4-R: | 5’ TAGTCGCTGCTTGATCGCTT 3’ | IGE GuangZhou |
| NANOG-F: | 5’ CCTGTGATTTGTGGGCCTGA 3’ | IGE GuangZhou |
| NANOG-R: | 5’ TGCGACACTCTTCTCTGCAG 3’ | IGE GuangZhou |
| SOX2-F: | 5’ GCTTAGCCTCGTCGATGAAC 3’ | IGE GuangZhou |
| SOX2-R: | 5’ AACCCCAAGATGCACAACTC 3’ | IGE GuangZhou |
| ALDH1-F: | 5’ CCGTGGCGTACTATGGATGC 3’ | IGE GuangZhou |
| ALDH1-R: | 5’ GCAGCAGACGATCTCTTTCGAT 3’ | IGE GuangZhou |
| Lin28-F: | 5’ TGCGGGCATCTGTAAGTGG 3’ | IGE GuangZhou |
| Lin28-R: | 5’ GGAACCCTTCCATGTGCAG 3’ | IGE GuangZhou |
| CyclinD1-F: | 5’ AATCCCATCACCATCTTCCA 3’ | IGE GuangZhou |
| CyclinD1-R: | 5’ CCTGCTTCACCACCTTCTTG 3’ | IGE GuangZhou |
| p27-F: | 5’ TGCAACCGACGATTCTTCTACTCAA 3’ | IGE GuangZhou |
| p27-R: | 5’ CAAGCAGTGATGTATCTGATAAACAAGGA 3’ | IGE GuangZhou |
| RPL39-F: | 5’ AgCgATTCCTggCCAAgAAA 3’ | IGE GuangZhou |
| RPL39-R: | 5’TCgTgACCTTCAgACAgCAT 3’ | IGE GuangZhou |
|  |  |  |

| **REAGENT or RESOURCE** | **SOURCE** | **IDENTIFIER** |
| --- | --- | --- |
| **Antibodies** |  |  |
| Rabbit polyclonal AGK | Abcam | Catalogue #:ab137616 |
| Mouse Monoclonal GAPDH | Proteintech | Catalogue #:60004-1-Ig |
| P21 Monoclonal antibody | Proteintech | Catalogue #:60214-1-lg |
| P27 (M-197) | Santa Cruz | Catalogue #:sc-776 |
| Cyclin D1 Monoclonal antibody | Proteintech | Catalogue #:60186-1-lg |
| Rb Polyclonal Antibody | Proteintech | Catalogue #:17218-1-AP |
| Anti-Rb (phospho S780) Antibody | Abcam | Catalogue #:ab47763 |
| Anti-alpha Tubulin | Abcam | Catalogue #:ab7291 |
| Rabbit polyclonal Ki-67 | Santa Cruz | Catalogue #:sc-15402 |
| Rabbit polyclonal IgG | Proteintech | Catalogue #:10284-1-AP |
| HRP- Goat Anti-Mouse IgG (H+L) | Proteintech | Catalogue #:SA00001-1 |
| HRP- Goat Anti-Rabbit IgG (H+L) | Proteintech | Catalogue #:SA00001-2 |
| Ribosomal Protein L39L antibody | Santa Cruz | Catalogue #:sc-100841 |
| MULK | Santa Cruz | Catalogue #:sc- sc-374390 |
| Tim22 Rabbit Polyclonal antibody | Proteintech | Catalogue #:25652-1-AP |
| Timm29 Rabbit Polyclonal antibody | Proteintech | Catalogue #:14927-1-AP |
| anti-OPA1 antibody | abcam | Catalogue #:ab157457 |
| recombinant anti-DRP1 antibody | abcam | Catalogue #:ab184247 |
| SOD-2 (E-10) | Santa Cruz | Catalogue #:sc-137254 |
| MFN1 | Santa Cruz | Catalogue #:sc-166644 |
| Ribosomal Protein L39 Antibody | LSBio | Catalogue #:LS-C385884-100 |
| VDAC1 Rabbit polyclonal antibody | Proteintech | Catalogue #:55259-1-AP |

**Supplemental Table 3. Antibodies used in this study.**

**Supplemental Table 4. Reagents used in this study.**

| **Reagent** | **Companies** | **Identifier** |
| --- | --- | --- |
| Cell Cycle Detection Kit | KeyGEN BioTECH | Catalogue #:KGA512 |
| PrimeScript ®RT reagent Kit with gDNA Eraser | Takara | Catalogue #:DRR047A |
| SYBR ® Premix Ex Taq TM II | Takara | Catalogue #:RR820A |
| TRIzol® Reagent | Invitrogen | Catalogue #:15596018 |
| DMEM basic (1×) | Gibco | Catalogue #:C11995500BT |
| Cell-Light™ EdU Apollo®567 In Vitro Imaging Kit | RIB BIO | Catalogue #:C10310-1 |
| KOD –Plus | TOYOBO | Catalogue #:KOD-201 |
| PCR Purification Kit (50) | QIAquick | Catalogue #:28104 |
| RIPA Tissue/cell lysis buffer | Solarbio | Catalogue #:R0020 |
| Plasmid extract kit (DP103) | TIANGEN | Catalogue #:DP103-03 |
| Lenti-Concentin Virus Precipitation solution | EXCell Bio | Catalogue #:EMB810A-1 |
| FBS | Gibco | Catalogue #:10091148 |
| QIAGEN Plasmid Maxi kit | QIAGEN | Catalogue #:12163 |
| seeblue plus2 pre-stained standard | Thermo | Catalogue #:LC5925 |
| Mitochondria lsolation Kit for Cultured Cells | Abcam | Catalogue #:ab110170 |
| Protein A/G PLUS-Agarose | Santa Cruz | Catalogue #:sc-2003 |
| mtDNA | TAKARA | Catalogue #:7246 |
| Respiratory Chain Complex I | Comin Biotechnology | Catalogue #:YX-031501H |
| Respiratory Chain Complex III | Comin Biotechnology | Catalogue #:YX-031503H |
| Respiratory Chain Complex IV | Comin Biotechnology | Catalogue #:YX-031504H |
| ATPase | Comin Biotechnology | Catalogue #:YX-031505H |
| ATP Assay Kit | BEYOTIME | Catalogue #:S0026 |
| DCFDA/H2DCFDA-cellular ROS Assay kit | Abcam | Catalogue #: ab113851 |
| mPTP Assay Kit | BEYOTIME | Catalogue #:C2009S |
| mito-Tracker | BEYOTIME | Catalogue #:C2006 |

**Supplemental Table 5. Laboratory apparatus used in this study**

| **Name** | **Company and Model** |
| --- | --- |
| Laser confocal microscope | OLYMPUS:FV1000 |
| Landing centrifuge | Japan's Hitachi:LR21N |
| Microporous plate luminous detector | Germany BERTHOLD:LB942 |
| Fluorescence quantitative PCR instrument | Germany Agilent:MX3000P |
| PCR instrument | Germany Agilent: sure cycles8800 |
| Gel imager | Tanon Science & Technology Co.,Ltd:Tanon-2500 |
| Uv-visible spectrophotometer | Japanese island ferry: UV-2550 |
| Carbon dioxide incubator | US Thermo: Forma3131 |
| Cryogenic refrigerator | Haier:DW-86L386 |
| Vertical electrophoresis tank | Tanon Science & Technology Co.,Ltd:VE-180 |
| Electrophoresis apparatus | Tanon Science & Technology Co.,Ltd: EPS-600+HE-120+VE |
| Inverted fluorescence study stage microscope | Japanese OLYMPUS:IX71+DP72+cellsense |
| Biosafety cabinet | SingaporeESCO:AC2-4S1 |
| Overspeed sorting flow cytometer | US BD: FACSAriaII |
