# Supplementary figures and images for: Acylglycerol kinase promotes ovarian cancer progression and regulates mitochondria function by interacting with ribosomal protein L39
Source: J Exp Clin Cancer Res. 2022 Aug 8;41:238. doi: 10.1186/s13046-022-02448-5 (PMC9358817; doi:10.1186/s13046-022-02448-5)

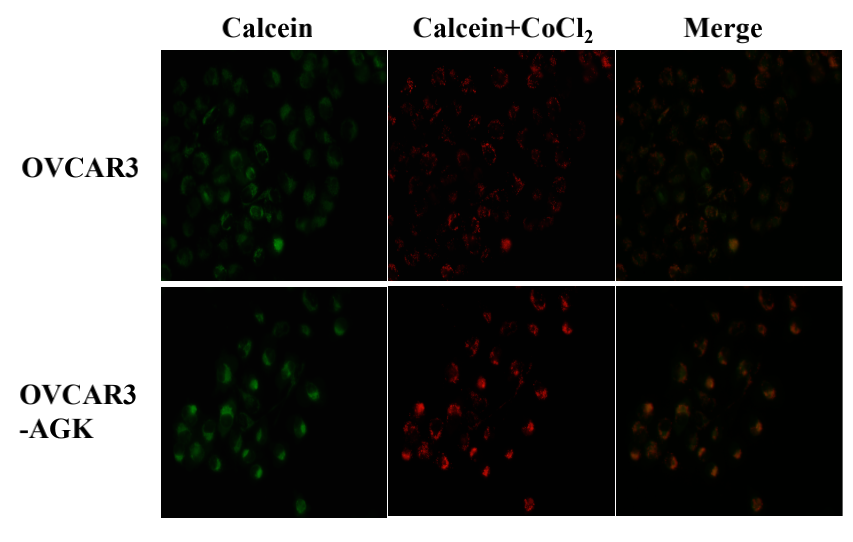

Supplement: Supplementary file 2 — Additional file 2: Supplemental Figure 1. AGK enhances the level of Δψm in OVCAR3 cells. [file 13046_2022_2448_MOESM2_ESM.tif]
